# Host Fitness: A Missing Dimension of Precision Medicine in Older Acute Myeloid Leukemia

**DOI:** 10.1002/hon.70249

**Published:** 2026-09-28

**Authors:** Fortunato Morabito, Antonella Bruzzese, Enrica Antonia Martino, Nicola Amodio, Ernesto Vigna, Massimo Gentile

**Affiliations:** ^1^ Dafne Srl Reggio Calabria Italy; ^2^ Department of Onco‐Hematology Hematology Unit AO of Cosenza Cosenza Italy; ^3^ Department of Experimental and Clinical Medicine University of Catanzaro Catanzaro Italy; ^4^ Department of Pharmacy, Health and Nutritional Science University of Calabria Rende Italy

**Keywords:** acute myeloid leukemia (AML), fitness, precision medicine

## Abstract

The therapeutic landscape for older adults with acute myeloid leukemia (AML) has undergone a major paradigm shift with the advent of lower‐intensity targeted regimens, particularly hypomethylating agents combined with venetoclax. As treatment transitions from acute induction to prolonged, multi‐cycle administration, clinical success increasingly depends on the patient's capacity to sustain repeated cycles, manage cytopenias and infections, and maintain functional independence. Current precision oncology models excel at characterizing the leukemic clone via cytogenetics, genomics, and measurable residual disease (MRD); however, they provide limited insight into the patient's underlying biological reserve. In this perspective, we propose *host fitness* as a critical, complementary dimension of precision medicine in older AML. Host fitness is a dynamic, multidimensional construct encompassing functional reserve, body composition, nutritional status, immune competence, inflammatory burden, and metabolic resilience. Moving beyond traditional performance status and simplistic metrics like body mass index, longitudinal assessment of host fitness aims to identify actionable vulnerabilities, guide targeted supportive interventions, and optimize treatment persistence. Integrating continuous host‐fitness evaluation with clonal profiling establishes a dual host–tumor precision‐medicine framework designed to maximize therapeutic efficacy while preserving patient resilience and quality of life.

## 1 Introduction

The management of older adults with acute myeloid leukemia (AML) has undergone a major therapeutic shift over the past decade. Historically, treatment decisions were driven primarily by whether a patient could tolerate intensive induction chemotherapy. Chronological age, performance status, comorbidity burden, and the clinician's global impression of “fitness” often determined treatment allocation, limiting access to disease‐modifying therapy for many older adults. The availability of lower‐intensity regimens, particularly hypomethylating agents (HMAs) combined with venetoclax, has changed this landscape by demonstrating that clinically meaningful remissions and survival benefit can be achieved in patients considered ineligible for intensive chemotherapy [1, 2].

This therapeutic evolution has also changed the determinants of clinical success. In intensive chemotherapy, early mortality, achievement of remission, and recovery from induction toxicity have traditionally dominated outcome assessment. By contrast, HMA–venetoclax therapy is usually delivered over multiple cycles and requires repeated management of cytopenias, infections, drug interactions, treatment interruptions, and dose or schedule modifications. Remission is therefore often the beginning, rather than the end, of successful treatment. The clinically relevant question is no longer only, “Can this patient start therapy?” but also, “Can this patient sustain treatment, recover from recurrent stressors, and remain well enough to derive durable benefit?” [3, 4].

This shift exposes a limitation of conventional risk assessment in older AML. Cytogenetic abnormalities, molecular alterations, and measurable residual disease (MRD) remain indispensable determinants of prognosis and treatment selection. However, these variables principally describe the behavior and therapeutic vulnerability of the leukemic clone. They provide only limited insight into the patient's capacity to tolerate sustained therapy, recover from infections or cytopenias, preserve function, and receive subsequent treatment cycles. In the venetoclax era, outcomes increasingly reflect the interaction between leukemia biology and the biological reserve of the host. Understanding this interaction may represent the next frontier of precision medicine in older AML.

Older adults with AML are highly heterogeneous. Patients of the same chronological age, with similar comorbidities and comparable leukemia‐risk profiles, may follow markedly different clinical trajectories because of differences in functional reserve, cognition, body composition, nutritional status, immune competence, and capacity to recover from physiological stress. Chronological age is therefore an incomplete surrogate for biological aging, while conventional performance‐status measures can overlook clinically meaningful vulnerabilities, including sarcopenia, malnutrition, cognitive impairment, and reduced physiological resilience [5, 6].

We propose that the next evolution of precision medicine in older AML should integrate characterization of the leukemic clone with systematic longitudinal assessment of the host. We refer to this complementary dimension as host fitness. We refer to this proposed construct as host fitness: a multidimensional and dynamic state reflecting the biological reserve required to tolerate treatment, recover from complications, preserve function, and sustain clinically effective therapy. Host fitness encompasses functional reserve, body composition, nutritional status, immune competence, inflammatory burden, and metabolic resilience. It is not intended as another binary “fit/unfit” label, nor as a tool to deny potentially effective treatment. Rather, it is a framework for identifying vulnerabilities that may be prognostically relevant, clinically actionable, and in some cases modifiable.

### 1.1 Host Fitness: A Complementary Dimension of Precision Medicine

Precision medicine in AML has traditionally focused on the biological characteristics of the malignant clone. Cytogenetics, molecular profiling, and increasingly sensitive MRD assessment have transformed prognostic classification and therapeutic decision‐making. These advances have enabled clinicians to identify disease subsets with distinct biology, response patterns, relapse risks, and therapeutic vulnerabilities [1].

However, a disease‐centered model captures only one component of the therapeutic equation. In older patients receiving prolonged lower‐intensity treatment, outcome depends not only on the sensitivity of leukemia to therapy but also on the capacity of the host to sustain repeated therapeutic exposure. A patient may have disease biology associated with a high likelihood of response and still fail to obtain durable benefit if infections, prolonged cytopenias, nutritional decline, functional deterioration, or loss of physiological reserve prevent adequate treatment delivery.

Host fitness is grounded in the recognition that aging is biologically heterogeneous. Two patients of the same age may differ substantially in mobility, independence, cognitive function, body composition, inflammatory state, immune reserve, and recovery capacity. These differences are not adequately captured by age alone, by comorbidity counts, or by a single performance‐status assessment. A multidimensional evaluation is therefore needed to understand the biological context in which leukemia‐directed therapy is delivered.

Frailty is among the most established manifestations of reduced host fitness. It is not simply the accumulation of chronic diseases, but a state of diminished physiological reserve and increased vulnerability to stressors. In older adults with cancer, including those with AML, geriatric assessment and frailty‐related measures identify vulnerabilities associated with toxicity, functional decline, and survival, providing information that complements conventional oncological risk stratification [6, 7, 8].

Frailty provides one of the strongest clinical foundations for the concept of host fitness, but it should not be considered synonymous with it. Rather, frailty represents a clinically observable manifestation of diminished biological reserve within a broader multidimensional construct that also includes body composition, nutritional status, immune competence, inflammatory burden, and metabolic resilience. Appreciating these complementary domains may help explain why patients with similar frailty scores experience markedly different capacities to tolerate and sustain therapy.

Among the biological domains not captured by frailty alone, body composition is particularly relevant because it provides an objective measure of physiological reserve. Although body mass index (BMI) is simple and universally available, it estimates body size rather than biological reserve. It cannot distinguish skeletal muscle from adipose tissue and therefore fails to identify clinically important phenotypes such as sarcopenia and sarcopenic obesity. Consequently, patients with similar BMI may differ substantially in muscle quality, inflammatory status, metabolic resilience, and capacity to tolerate treatment. In cancer populations, low skeletal‐muscle mass and sarcopenic obesity have been associated with treatment toxicity, complications, impaired functional reserve, and poorer survival [9, 10].

Nutritional status is another essential domain. Nutritional impairment should not be understood simply as low body weight. In patients with cancer, it may reflect reduced dietary intake, systemic inflammation, altered metabolism, catabolism, impaired tissue maintenance, and diminished capacity for immune recovery. Contemporary definitions of malnutrition integrate phenotypic criteria, such as weight loss or reduced muscle mass, with etiologic factors, including inflammation and disease burden [11].

Host fitness also includes immune, inflammatory, and metabolic domains. Aging is associated with immunosenescence, chronic low‐grade inflammation, and impaired regenerative capacity, processes that may influence susceptibility to infection and recovery from treatment‐related complications. The relevance of these mechanisms to AML treatment persistence is biologically plausible, but they should currently be regarded as priorities for prospective investigation rather than as validated clinical determinants of treatment selection [12, 13].

Host fitness should therefore not be regarded as a collection of unrelated variables or as a new static eligibility score. It is an integrated and dynamic state resulting from the interaction of functional capacity, body composition, nutritional resources, immune competence, inflammatory regulation, metabolic adaptation, leukemia burden, and treatment exposure. The relevant question is not whether a patient is categorically “fit” or “unfit,” but how much biological reserve is available, which deficits are present, how rapidly they are changing, and whether targeted intervention can preserve the capacity to benefit from therapy.

In this framework, leukemia genomics defines the vulnerabilities of the malignant clone, whereas host fitness defines the capacity of the individual to exploit available therapeutic opportunities. Precision medicine in older AML should therefore evolve toward a dual biological model in which leukemia biology defines therapeutic opportunity and host fitness defines the capacity to exploit it.

### 1.2 Treatment Resilience and Treatment Persistence in the Venetoclax Era

The introduction of venetoclax‐based regimens has changed both the therapeutic options available to older adults with AML and the meaning of treatment success. With intensive chemotherapy, outcome has often been determined by early events: remission induction, early mortality, and recovery from acute toxicity. HMA–venetoclax regimens represent a different paradigm. They can induce deep remissions in patients unsuitable for intensive therapy, but their benefit frequently depends on repeated cycles, individualized schedule modification, infection prevention, and management of prolonged myelosuppression [2, 5].

We define treatment resilience as the capacity to tolerate repeated therapeutic stress while retaining sufficient biological reserve to continue clinically effective treatment. Unlike a baseline eligibility decision, treatment resilience is dynamic. It evolves in response to leukemia burden, remission status, cumulative myelosuppression, infectious complications, hospitalization, nutritional deterioration, functional decline, and the effectiveness of supportive interventions.

A related concept, treatment persistence, should become an operational construct in future studies.

Treatment persistence represents the longitudinal clinical expression of host fitness. Whereas host fitness reflects the biological reserve available to tolerate therapy, treatment persistence reflects whether that reserve is sufficient to sustain clinically effective treatment over time.

Real‐world experience has emphasized that treatment delivery in older AML is frequently complicated by prolonged cytopenias, infectious events, hospitalizations, and treatment modifications. These events are not simply logistical obstacles. They may determine whether an otherwise active antileukemic regimen can be administered for long enough to generate durable benefit [14, 15, 16, 17, 18].

Infectious complications provide a clear example of the interaction between host biology and therapeutic efficacy. Older patients with AML are vulnerable to infection because of leukemia‐related immune dysfunction, treatment‐induced neutropenia, age‐associated immune decline, comorbidities, and impaired physiological reserve. The consequences extend beyond acute morbidity: infection can cause hospitalization, treatment delay, functional loss, nutritional deterioration, dose modification, and premature treatment discontinuation. Infection prevention and rapid management should therefore be viewed not only as supportive‐care measures but also as components of effective treatment delivery [19, 20].

Similarly, cytopenias should not be interpreted solely as a pharmacological consequence of therapy. Their duration and clinical impact are likely influenced by baseline marrow reserve, leukemia control, prior therapy, nutritional state, inflammatory activity, infection, and overall physiological resilience. This does not mean that each of these factors is already validated as an independent predictor of treatment tolerance in HMA–venetoclax‐treated AML. It does mean that strategies to optimize outcomes should consider both appropriate adaptation of antileukemic treatment and preservation of host capacity.

The relationship between host fitness and MRD is especially important. MRD has become one of the most powerful measures of response depth and relapse risk in AML. In patients treated with azacitidine–venetoclax, MRD negativity is associated with favorable clinical outcomes [21, 22]. Yet MRD and host fitness capture distinct dimensions of outcome. MRD reflects residual leukemic burden and therapeutic sensitivity, whereas host fitness reflects the patient's capacity to tolerate continuing therapy and recover from its complications.

Patients with comparable MRD status may therefore have different treatment trajectories. A patient with persistent MRD but preserved function and physiological reserve may remain eligible for additional treatment cycles or response‐deepening strategies. Another patient with a similar disease burden but progressive sarcopenia, recurrent infection, or functional decline may require treatment adaptation, intensified supportive care, or a reassessment of therapeutic goals. The clinical value of host‐fitness assessment lies not in replacing MRD or molecular risk, but in providing the complementary information required to interpret whether a biologically effective strategy is clinically sustainable.

The optimal treatment strategy in older AML may therefore not be the regimen with the greatest antileukemic activity in isolation. It may be the strategy that achieves the most favorable balance between leukemia control, treatment exposure, toxicity management, and preservation of host capacity. In this perspective, supportive care is not ancillary to precision therapy; it is part of the strategy that enables precision therapy to succeed.

### 1.3 Beyond BMI: Why Conventional Measures Fail to Capture Biological Reserve

BMI illustrates the limitations of conventional measures of host fitness. Although simple and universally available, BMI reflects body size rather than biological reserve and cannot distinguish skeletal‐muscle mass from adipose tissue or capture muscle quality, metabolic dysfunction, inflammatory burden, or functional capacity. This limitation is particularly relevant in older AML, where aging, disease‐related inflammation, reduced activity, and treatment exposure may promote muscle loss despite stable or increased body weight. Thus, patients with similar BMI may have markedly different physiological reserve and capacity to tolerate therapy.

Sarcopenia is defined by progressive loss of skeletal‐muscle mass and function associated with aging and disease. It represents a specific biological component of reduced host fitness, reflecting not only loss of muscle quantity but also impaired muscle quality and functional reserve. In cancer populations, sarcopenia and sarcopenic obesity have been associated with increased treatment toxicity, complications, impaired functional recovery, and poorer survival [9, 23].

In AML, prospective evidence supporting body‐composition assessment as a treatment‐selection tool remains limited. However, body composition may identify vulnerabilities not captured by age, ECOG performance status, comorbidity burden, or BMI and should therefore be considered a priority area for prospective validation rather than a criterion for treatment exclusion [24].

Routine CT imaging performed during AML evaluation may provide an opportunity for objective body‐composition assessment without additional procedures. However, before integration into clinical practice, prospective studies are needed to define standardized measures, clinically relevant thresholds, and whether CT‐derived parameters improve treatment decision‐making beyond established assessments [25, 26].

The central lesson extends beyond BMI. No single parameter can adequately capture host fitness. Performance status, frailty measures, nutritional screening, inflammatory biomarkers, body composition, and functional tests each describe different aspects of reserve. The goal should not be to replace BMI with another isolated biomarker, but to develop multidimensional models that integrate complementary domains and identify modifiable vulnerabilities.

Historically, treatment selection in older AML was guided largely by age, performance status, and comorbidity burden. Precision oncology subsequently emphasized genomic alterations, molecular‐risk categories, and MRD kinetics. Neither approach fully captures the contemporary challenge of delivering prolonged, lower‐intensity treatment. A host–tumor precision‐medicine model integrates these perspectives: leukemia biology identifies what treatment may work, while host fitness helps determine whether that treatment can be delivered safely, consistently, and effectively.

Host fitness should be assessed longitudinally. Baseline assessment remains important, but a single pre‐treatment evaluation cannot capture changes induced by leukemia, remission, infection, hospitalization, treatment toxicity, inactivity, or aging‐related decline. A patient who appears robust at diagnosis may develop marked functional or nutritional deterioration during therapy; conversely, improved leukemia control and effective supportive interventions may restore aspects of reserve.

This concept parallels the evolution of MRD assessment. Response assessment has moved from binary categories, such as remission or relapse, to a dynamic measurement of disease burden over time. Similarly, host fitness should be regarded as a continuum that can improve, remain stable, or deteriorate during the disease course. Longitudinal evaluation could identify early loss of resilience and create an opportunity for intervention before functional decline becomes irreversible.

Importantly, the purpose is not to apply uniform interventions to every patient. Host‐fitness assessment should identify the domain most likely to limit treatment delivery. A patient with impaired mobility and muscle loss may need physical rehabilitation and resistance exercise; a patient with nutritional vulnerability may need dietitian‐led intervention; a patient with recurrent infection may require individualized prophylactic and monitoring strategies; and a patient with substantial polypharmacy or cognitive impairment may require medication review and caregiver support. This is precision supportive care within a precision‐medicine framework.

### 1.4 From Precision Oncology to Host–Tumor Precision Medicine

The success of precision medicine in AML has been driven by increasingly refined characterization of leukemia biology. Cytogenetic classification, molecular profiling, and MRD assessment have transformed AML from a morphologically defined disease into a biologically heterogeneous group of disorders requiring individualized management [1].

The next phase of precision medicine should not abandon this disease‐centered progress. Rather, it should complement it with a structured assessment of the host. Molecular abnormalities define therapeutic sensitivity and risk of resistance; MRD reflects response depth and residual disease burden; host fitness describes the biological capacity to sustain therapy and recover from treatment‐associated stress. These dimensions are complementary, and their interaction may explain why patients with similar molecular risk and comparable early responses experience divergent long‐term outcomes.

The conceptual evolution proposed here is summarized in Figure 1. Traditional assessment in older AML was designed principally to determine treatment eligibility. Precision oncology then refined prediction of leukemia response through cytogenetic, molecular, and MRD‐based characterization. We propose a complementary host–tumor model in which leukemia biology and host fitness are evaluated together to estimate not only the likelihood of response, but also the likelihood that effective therapy can be sustained.

**FIGURE 1:**
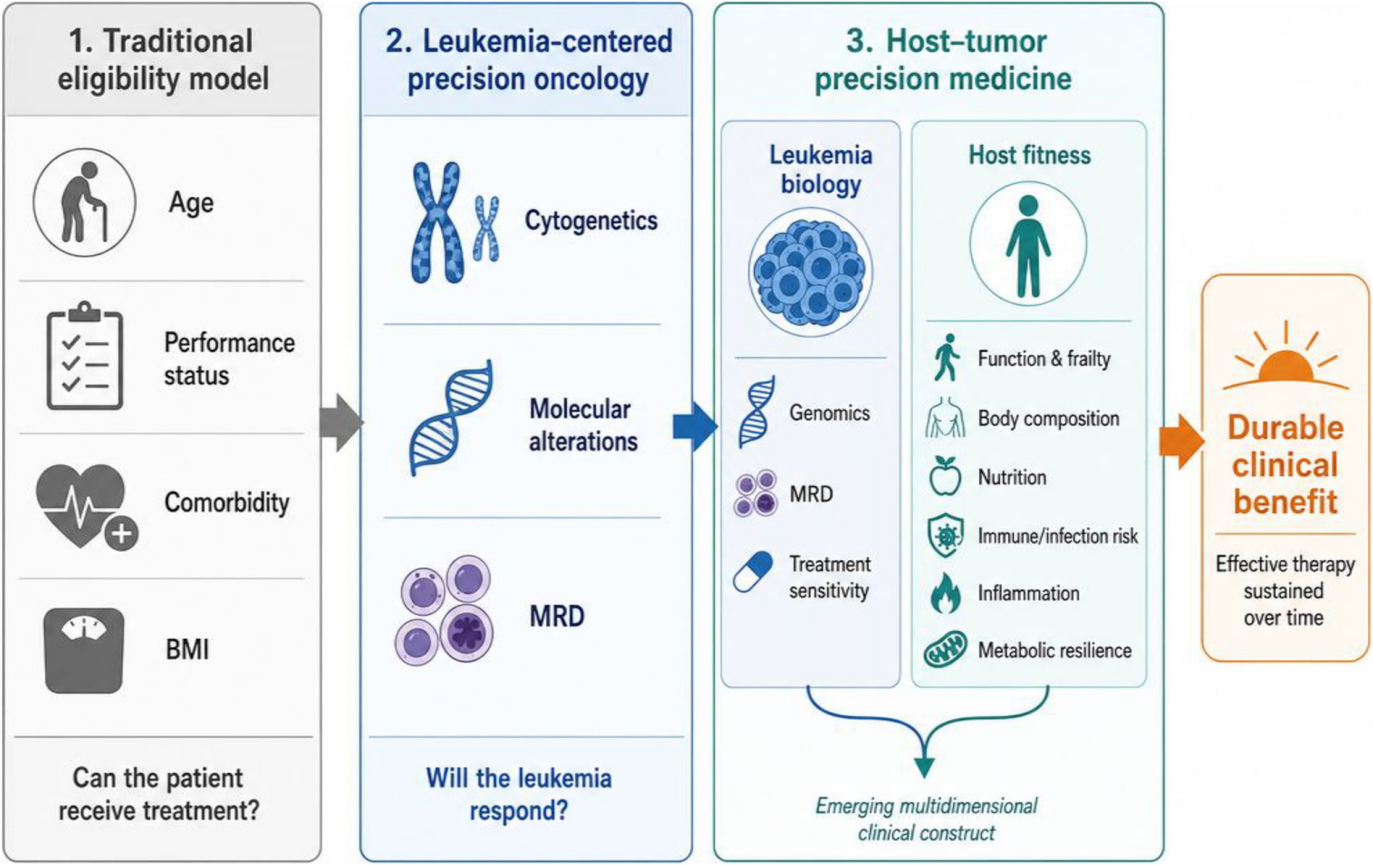
From treatment eligibility to host–tumor precision medicine in older AML. Traditional treatment selection in older AML has relied predominantly on chronological age, performance status, comorbidity burden, and body size to estimate whether intensive treatment is feasible. Precision oncology subsequently introduced cytogenetic, molecular, and measurable residual disease (MRD) assessments to define leukemia biology and therapeutic sensitivity. The proposed host–tumor precision‐medicine model integrates leukemia‐directed variables with longitudinal assessment of host fitness. In this model, leukemia biology informs the likelihood of response, whereas host fitness reflects the capacity to tolerate treatment, recover from complications, and sustain clinically effective therapy.

Figure 1 summarizes the central hypothesis of this perspective: precision medicine in older aml should evolve from a predominantly leukemia‐centered model toward an integrated host–tumor framework. Cytogenetic abnormalities, molecular alterations, and mrd characterize the biological behavior, therapeutic sensitivity, and residual burden of the leukemic clone. Host fitness provides the complementary information required to understand whether a patient can tolerate treatment, recover from complications, and sustain clinically effective therapy. Treatment outcome is therefore determined not only by selection of the most appropriate antileukemic strategy, but also by the interaction between leukemia sensitivity and the biological reserve of the host [1, 21].

The concept of host fitness is therefore an extension—not a replacement—of precision oncology. Leukemia genomics identifies disease‐specific vulnerabilities, whereas host fitness describes the patient's capacity to exploit available therapeutic opportunities. Integrating these dimensions may help explain why patients with comparable molecular‐risk profiles, similar remission depth, or equivalent MRD status may nevertheless experience markedly different trajectories with respect to toxicity, hospitalization, treatment exposure, functional decline, and durable disease control.

Host fitness should not be considered a binary classification of “fit” versus “unfit” patients. Rather, it should be regarded as a dynamic continuum that changes during the disease course in response to leukemia burden, treatment exposure, cytopenias, infections, hospitalization, nutritional decline, physical inactivity, and aging‐related processes. This longitudinal perspective parallels the evolution of MRD assessment from a binary response measure to a dynamic indicator of disease burden. Repeated assessment of host fitness may allow early identification of declining treatment resilience and provide an opportunity for targeted supportive interventions or treatment adaptation before functional deterioration becomes irreversible [6, 27].

This framework also challenges the traditional separation between antileukemic therapy and supportive care. The objective is not simply to classify vulnerability, nor to apply identical interventions to all patients, but to identify the specific and potentially modifiable factors that limit treatment delivery. A patient with sarcopenia may require targeted rehabilitation and nutritional intervention, whereas recurrent infections, medication burden, functional decline, or social vulnerability may require different strategies. As summarized in Table 1, host fitness is an integrated construct comprising functional reserve, body composition, nutritional status, immune competence, inflammatory burden, and metabolic resilience. These domains should not be used to create another static eligibility score, but to guide individualized strategies that preserve the capacity of older adults with AML to receive and benefit from sustained antileukemic therapy.

**TABLE 1:** Domains of host fitness and their potential implications for treatment resilience in older adults with acute myeloid leukemia.

Host‐fitness domain	Information captured	Potential relevance in AML treatment	Practical assessment at baseline and during therapy	Potential clinical actions and research priorities
Functional reserve and frailty	Mobility, physical performance, independence in activities of daily living, cognition, falls, psychological status, social support, and vulnerability to physiological stress	Identifies patients at risk of treatment toxicity, hospitalization, functional decline, loss of independence, and inability to sustain planned therapy	Comprehensive geriatric assessment or validated abbreviated screening; ECOG performance status; activities and instrumental activities of daily living; gait speed; chair‐stand testing; falls history; cognitive screening	Geriatric co‐management; physical therapy; occupational therapy; medication review; fall prevention; caregiver and social‐support interventions; prospective validation of AML‐specific longitudinal functional endpoints
Body composition	Skeletal‐muscle mass and quality, adipose distribution, sarcopenia, sarcopenic obesity, and weight trajectory	May identify hidden vulnerability not captured by chronological age, performance status, comorbidity burden, or BMI; may influence recovery from treatment‐related stress and functional decline	Weight change; nutritional examination; opportunistic CT‐based assessment of skeletal‐muscle area and adipose tissue when routine imaging is available; serial functional measures	Dietitian and rehabilitation referral; resistance and mobility interventions where feasible; prospective validation of CT‐derived thresholds, optimal timing, and clinical utility in AML
Nutritional status	Dietary intake, involuntary weight loss, reduced muscle mass, nutritional symptoms, inflammation‐associated malnutrition, and capacity for tissue maintenance and recovery	Nutritional impairment may contribute to reduced physiological reserve, impaired recovery, infection vulnerability, treatment interruption, and functional deterioration	Validated nutritional screening; weight‐loss history; dietary‐intake assessment; nutrition‐focused physical examination; GLIM‐based assessment when appropriate	Early dietitian‐led intervention; oral nutritional supplementation; symptom management; enteral or parenteral support when clinically indicated; trials testing effects on function, treatment exposure, and quality of life
Immune competence and infection vulnerability	Prior infections, immune recovery, neutropenia, immunosenescence, exposure risk, vaccination status, and capacity to recover after infection	Influences susceptibility to bacterial, viral, and fungal infection; infections may cause hospitalization, functional decline, treatment delays, and early discontinuation	Infection history; baseline and serial blood counts; assessment of prior invasive fungal infection; medication and drug‐interaction review; monitoring during prolonged neutropenia	Individualized antimicrobial prophylaxis and surveillance according to institutional and guideline‐based practice; prompt diagnostic evaluation and treatment of infection; prospective development of immune‐risk models in HMA–venetoclax‐treated AML
Inflammatory burden	Systemic inflammation, chronic inflammatory conditions, acute infection, disease‐associated catabolism, and biological aging‐related inflammatory activation	May contribute to nutritional decline, muscle loss, impaired recovery, and reduced physiological reserve; its independent predictive role in AML requires validation	Clinical assessment for infection and inflammatory comorbidity; routine laboratory markers when clinically indicated; longitudinal interpretation in the context of leukemia activity and treatment	Treat reversible inflammatory drivers; integrate with nutritional and functional assessment; prospective studies to identify robust, actionable inflammatory biomarkers of treatment resilience
Metabolic resilience	Glucose regulation, renal and hepatic reserve, energy homeostasis, metabolic adaptation to stress, and potentially mitochondrial function	May influence tolerance of treatment, recovery from illness, medication handling, and capacity to adapt to prolonged therapeutic stress; currently an emerging research domain	Routine renal, hepatic, electrolyte, and metabolic evaluation; assessment of diabetes, cardiovascular disease, and other metabolic comorbidities; serial monitoring during therapy	Optimization of reversible metabolic abnormalities and comorbidities; medication adjustment; prospective validation of metabolic and mitochondrial biomarkers before routine use
Treatment persistence, exposure, and recovery trajectory	Cycle delivery, treatment interruptions, dose or schedule modifications, cytopenia recovery, hospitalization burden, transfusion dependence, and patient‐reported function	Provides a pragmatic longitudinal measure of treatment resilience and may help distinguish appropriate treatment adaptation from clinically limiting toxicity	Number and timing of completed cycles; duration of cytopenias; unplanned delays; hospitalization days; transfusion needs; patient‐reported outcomes; functional trajectory	Individualized HMA–venetoclax schedule adaptation; optimization of supportive care; early rehabilitation after hospitalization; incorporation of treatment persistence and function‐preserving endpoints into prospective clinical trials

Abbreviations: AML, acute myeloid leukemia; BMI, body mass index; CT, computed tomography; ECOG, Eastern Cooperative Oncology Group; GLIM, Global Leadership Initiative on Malnutrition; HMA, hypomethylating agent.

### 1.5 Future Directions: Measuring and Improving Host Fitness

Translating host fitness into clinical practice requires that it become measurable, reproducible, and clinically useful. At present, host‐related factors are often evaluated qualitatively through physician judgment, performance status, isolated laboratory values, or selected elements of geriatric assessment. These approaches provide valuable information, but they do not adequately capture the multidimensional and dynamic nature of host reserve.

A practical first step is systematic incorporation of geriatric assessment into the management of older adults with AML. Comprehensive geriatric assessment evaluates function, cognition, psychological health, social support, comorbidity, medication burden, and nutritional vulnerability. In oncology, it identifies deficits not evident from routine performance‐status assessment and helps predict treatment‐related toxicity, functional decline, and survival [7, 27].

In AML practice, a full geriatric assessment may not always be feasible at diagnosis. A staged approach may therefore be more realistic. An initial screen could include performance status, activities of daily living, gait speed or chair‐stand testing, cognition, falls, medication review, nutritional risk, social support, and baseline weight change. Patients with identified vulnerabilities could then undergo more comprehensive assessment and targeted referral. This approach should complement, rather than delay, timely initiation of leukemia‐directed therapy.

CT‐based body‐composition analysis is another promising approach because CT imaging is often already performed as part of AML care. It can provide objective information on skeletal‐muscle mass, muscle radiodensity, and adipose distribution without additional procedures [24]. In AML, however, prospective evidence that CT‐derived metrics improve risk prediction, guide intervention, or alter clinical outcomes beyond standard assessment remains limited. Automated image‐analysis approaches may facilitate implementation and longitudinal monitoring, but technology should follow—not substitute for—clinical validation [26].

Inflammatory, immune, and metabolic biomarkers may eventually add biological depth to host‐fitness profiling. At present, however, their optimal selection, timing, thresholds, reproducibility, and clinical utility remain uncertain. Future studies should prioritize feasible measures that can be repeatedly obtained in routine care and should test whether they improve prediction beyond established clinical and geriatric variables.

The clinical relevance of host fitness will depend not only on prognostic discrimination but also on actionability. Several domains are potentially modifiable. Nutritional intervention, exercise or rehabilitation, optimization of comorbidities, medication review, infection prevention, transfusion support, early physical therapy, and social‐support strategies may preserve aspects of host capacity. Direct evidence that these interventions improve survival or treatment persistence specifically in HMA–venetoclax‐treated AML remains limited; this should be stated clearly. Nevertheless, their effects on function, symptoms, nutritional status, and quality of life make them appropriate candidates for prospective evaluation.

Clinical trials in older AML should extend beyond conventional disease‐centered endpoints. Response rate, MRD negativity, event‐free survival, and overall survival remain fundamental, but they do not fully capture whether treatment is sustainable or whether clinical benefit is achieved at the cost of substantial functional decline.

Future clinical trials should incorporate host fitness variables prospectively, not only as covariates but as clinically meaningful endpoints that complement established measures of disease response and survival. These host‐related endpoints should be evaluated alongside, rather than replace, conventional disease‐related outcomes, including response depth, MRD assessment, and long‐term disease control. Such an approach would allow trials to capture not only whether a therapy can eradicate leukemia, but also whether patients can tolerate, sustain, and derive meaningful benefit from treatment over time. The most effective therapy is therefore not only the one that achieves disease control, but the one that can be successfully delivered within the biological capacity of the patient receiving it.

Interventional trials should determine whether targeted strategies aimed at improving host fitness can modify treatment delivery and patient‐centered outcomes. Potential approaches may include early nutritional interventions, structured exercise and rehabilitation programs, intensified infection‐prevention strategies, and integrated geriatric co‐management. The objective is not to delay or replace antileukemic therapy, but to optimize the biological and functional conditions required for effective treatment delivery and sustained benefit.

A future AML‐care model could therefore involve parallel longitudinal monitoring of leukemia and host biology. Molecular profiling and MRD assessment would characterize disease evolution and therapeutic sensitivity, while functional status, nutritional risk, body composition, infection burden, treatment exposure, and patient‐reported outcomes would define the patient's capacity to meet therapeutic demands. Treatment adaptation could then respond dynamically to changes in both disease biology and host reserve.

## 2 Conclusions

The therapeutic landscape of older AML has reached an important transition point. Effective lower‐intensity regimens, particularly HMA–venetoclax combinations, have challenged the assumption that advanced age or comorbidity alone should define therapeutic limitation. Yet, as remission becomes achievable for more older adults, the determinants of durable benefit are changing. The central challenge is therefore shifting from identifying patients who can initiate therapy to identifying and supporting those who can sustain effective treatment long enough to derive meaningful benefit.

Leukemia biology remains indispensable. Cytogenetic risk, molecular alterations, and MRD dynamics define the behavior and vulnerabilities of the malignant clone. However, these factors do not fully explain why patients with similar disease features and comparable initial responses can experience markedly different outcomes. Host fitness provides a complementary framework for understanding why patients with similar disease characteristics may differ in their ability to tolerate treatment, recover from complications, and sustain therapeutic benefit.

Host fitness should not be treated as a static label or a reason to withhold treatment. It is a dynamic and potentially modifiable state that integrates functional reserve, body composition, nutritional status, immune competence, inflammatory burden, and metabolic resilience. Its clinical value lies in identifying vulnerabilities that are potentially modifiable and can inform individualized supportive strategies and treatment adaptation.

As illustrated in Figure 2, host‐fitness assessment should extend beyond a single evaluation at diagnosis and become integrated into the therapeutic pathway. Baseline assessment may identify functional, nutritional, body‐composition, infectious, cognitive, and social vulnerabilities, while longitudinal monitoring may detect changes in biological reserve that require supportive intervention or treatment adaptation. This approach creates opportunities to preserve treatment feasibility before declining host capacity compromises the ability to continue effective antileukemic therapy.

**FIGURE 2:**
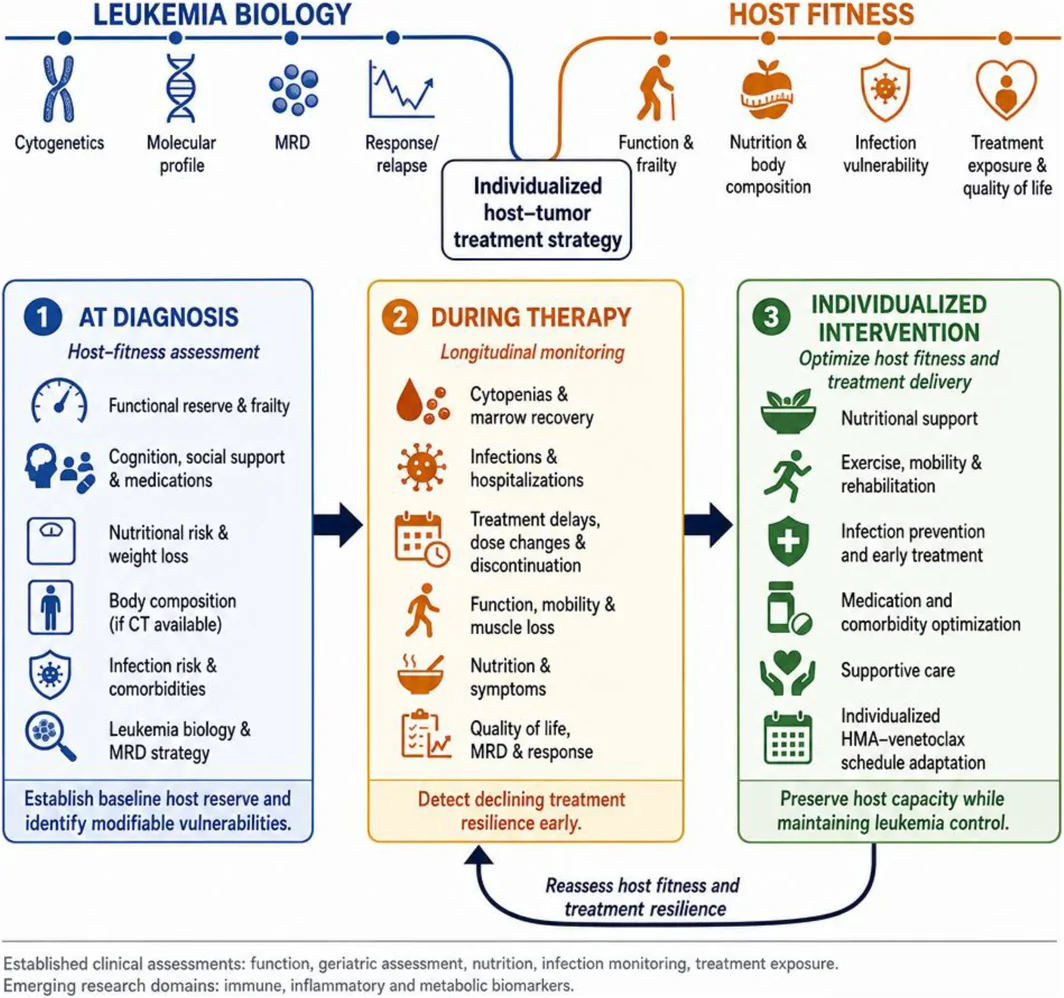
Longitudinal assessment and optimization of host fitness during treatment of older adults with acute myeloid leukemia. Host fitness should be evaluated at diagnosis and reassessed throughout treatment rather than regarded as a single baseline eligibility determination. At diagnosis, assessment integrates functional reserve and frailty, cognition, social support, medication burden, nutritional risk, body composition when clinically available, infection vulnerability, comorbidities, and leukemia‐specific variables, including cytogenetic and molecular risk assessment and the planned measurable residual disease (MRD) monitoring strategy. During therapy, longitudinal monitoring captures changes in cytopenia recovery, infectious complications, hospitalization burden, treatment delays or schedule modifications, functional status, mobility, nutritional state, body composition, quality of life, and leukemia response. Declining treatment resilience may prompt individualized interventions, including nutritional support, rehabilitation and exercise strategies, infection prevention and early treatment, optimization of comorbidities and medication burden, supportive‐care measures, and adaptation of HMA–venetoclax delivery. Integration of host‐fitness monitoring with leukemia biology and MRD assessment provides a dynamic host–tumor precision‐medicine framework aimed at preserving host capacity while maintaining effective leukemia control.

The next generation of studies in older AML should test whether systematic, longitudinal assessment of host fitness improves clinically meaningful outcomes, including treatment persistence, hospitalization burden, functional preservation, quality of life, and survival. Such studies should distinguish established clinical measures from exploratory biomarkers and should evaluate interventions directed at modifiable vulnerabilities.

True precision medicine in older AML will require a dual biological map: one describing the leukemia and one describing the host. Integrating these dimensions may move the field beyond a model focused solely on matching therapies to leukemia characteristics toward a host–tumor precision‐medicine strategy that also maximizes the patient's capacity to receive, sustain, and benefit from treatment. In the venetoclax era, understanding the host may become as important as understanding the leukemia itself.

## Author Contributions

All authors contributed to the manuscript and were involved in revisions and proofreading. All authors approved the submitted version.

## Funding

The authors have nothing to report.

## Ethics Statement

The authors have nothing to report.

## Conflicts of Interest

The authors declare no conflicts of interest.

## Data Availability

Data sharing does not apply to this article, as no new data were generated or analyzed in this study.
